# On the Forward Scattering of Microwave Breast Imaging

**DOI:** 10.1155/2012/582037

**Published:** 2012-05-06

**Authors:** Hoi-Shun Lui, Andreas Fhager, Mikael Persson

**Affiliations:** ^1^Biomedical Electromagnetics Research Group, Division of Signal Processing and Biomedical Engineering, Department of Signals and Systems, Chalmers University of Technology, S 412 96 Gothenburg, Sweden; ^2^Medtech West, Sahlgrenska University Hospital, SE413-45 Gothenburg, Sweden

## Abstract

Microwave imaging for breast cancer detection has been of significant interest for the last two decades. Recent studies focus on solving the imaging problem using an inverse scattering approach. Efforts have mainly been focused on the development of the inverse scattering algorithms, experimental setup, antenna design and clinical trials. However, the success of microwave breast imaging also heavily relies on the quality of the forward data such that the tumor inside the breast volume is well illuminated. In this work, a numerical study of the forward scattering data is conducted. The scattering behavior of simple breast models under different polarization states and aspect angles of illumination are considered. Numerical results have demonstrated that better data contrast could be obtained when the breast volume is illuminated using cross-polarized components in linear polarization basis or the copolarized components in the circular polarization basis.

## 1. Introduction

In the last two decades, extensive studies have been contributed to breast cancer detection using microwave-based technologies [1–25]. Compared to X-ray mammography that is widely used in hospital nowadays, microwave is a nonionizing radiation which is safer to subjects. Early studies [3–5] reported that there is a significant contrast between malignant tumor and healthy breast tissue which forms a strong foundation for the use of microwave-based techniques for breast cancer detection, although recent studies [26, 27] found that the contrast is much lower. This raises an issue of whether malignant tumor can be detected, especially when the relative permittivity of the tumor is close to that of the surrounding tissue.

In general, studies of microwave breast cancer detection can be divided mainly into two main groups, namely, the radar-based imaging approach (e.g., [1–12]) and the inverse scattering approach [13–20]. Radar-based imaging approach, first proposed by Hagness et al. [3–5], aims to identify the presence and location of strong scatterers due to the significant contrast between the tumor and the healthy breast tissue. This involves focusing on reflections from the breast, that is, a coherent-sum process adapted from synthetic aperture radar techniques [4]. Throughout the years, numerous studies have been conducted from different research groups [6–12] and variations of the original radar-based technique, such as microwave imaging via space-time (MIST) beamforming [6, 7], tissue sensing adaptive radar (TSAR) [8, 9] have been proposed.

Here, we mainly focus on the inverse scattering approach [13–20]. The objective of inverse scattering is to reconstruct the unknown dielectric profiles of the breast volume. First, multistatic measurement from the breast volume is taken as reference. Based on numerical solutions of the Maxwell's equations (e.g., finite element method [13, 14], method of moment [15], finite-difference time-domain (FDTD) [16–18]), the entire volume can be spatially discretized into a number of variables with unknown dielectric properties. The corresponding forward problems with the same transmitter/receiver configuration are included and the computation can be done with an initial guess of the dielectric profiles of the breast volume. Given the reference data from the “actual” breast volume and the simulated data from the “assumed” breast volume, a cost function based on the differences between these two datasets is defined. The cost function is then minimized in an iterative manner by changing the dielectric properties in the modeling domain using a gradient-based optimization algorithm. Assuming that the global minima is reached at the end of the optimization process, that is, the simulated data is almost the same or even identical to the reference data, the resulting dielectric profiles in the simulation domain is thus the resultant image provide that the global minima is reached.

The image reconstruction process is an ill-posed multidimensional optimization problem that the number of unknown variables (*ε*_*r*_(*x*, *y*, *z*), *σ*(*x*, *y*, *z*)) is determined in the image reconstruction stage. The number of unknown variables depends mainly on the physical size, the dielectric properties and geometry of the object, the required spatial resolution, and the frequency of interest (which is also a factor that determines the spatial discretization in the computation domain). Optimization of such a high-dimensional problem with thousands unknown variables is not trivial and chances for trapping into local minima could be high.

Furthermore, the uniqueness of the solution is also important. Given a set of measured-referenced data, there could be more than one distribution of dielectric profiles that can result in the same or similar measured data. As an example, if we consider some earlier studies in the literature [21] that compares the monostatic ultra wideband (UWB) response from the breast volumes with and without tumor, it is found that the differences between the copolarized components of the two cases is lower than that of the cross-polarized components. In other words, it is difficult to tell if there is any tumor by looking at the copolarized response, but the contrast is more apparent if the cross-polarized response is considered.

Polarization diversity has been widely used for radar imaging. The transmitters and receivers are located in the far-field region such that higher-order interactions between the object and the transmitters, as well as that of the object and the receivers, can be ignored. Coupling between transmitting and receiving antennas can be also ignored and thus the measured responses are purely object dependent. In microwave breast imaging scenario, the breast volume is surrounded by an antenna array in the near-field region. Mutual coupling between antenna elements as well as higher-order interactions between the breast volume and the antennas is significant. To reduce the reflection from the skin-air interface, the breast volume is usually immersed into some matching liquid. A possible exemption could be the case if the operation frequency is high enough such that the antenna is electromagnetically far from the breast and the conductivity of the matching liquid is relatively high such that higher-order interactions are attenuated. In microwave tomography, monopole antenna elements are used and thus only linear copolarized responses of the breast volume are considered in the image reconstruction process [15, 16].

In this paper, the forward scattering data of the two cases, that is, breast volume with and without tumor, under different polarization basis throughout the entire frequency bandwidth is investigated. The objective here is to investigate if better contrast of the forward data can be obtained such that the two cases are more distinguishable. With two distinguishable sets of forward data, chances for the two inverse problems heading to the same solution in the optimization process could be reduced. To simplify the analysis, homogenous breast volume together with scattered far-field under different polarization basis is considered. We are aware that early efforts have been contributed to issues such as array configurations, development of image reconstructions and efficient forward solvers [25]. A summary of microwave inverse scattering can be found in [28]. We are also well aware that recent efforts have been contributed to experimental setups and clinical studies of microwave breast imaging, for instance the special session that was conducted in the IEEE Antenna and Propagation Society Conference in 2010 in Toronto [22]. This is also one of the major research topics in the research group in Chalmers. A dedicated, high-performance UWB time domain microwave breast imaging system is under development [23, 24]. The reasons behind such simplified models and setup considered in this work are given as follows.

First, we would like to see if the scattering problem itself under a different polarization basis resulted in forward data with better contrast that can be used for solving the inverse problem. Here, contrast means the differences of the forward data between the two cases: breast volume (i) with and (ii) without tumor. Chances for the two problems heading towards the same local minima could probably be reduced if the contrast is high. In electromagnetic scattering, any object can be treated as a polarization transformer that depolarizes the incoming electromagnetic wave. Boerner et al. [29] pointed out the importance of having full polarimetric data when the electromagnetic inverse problem is formulated. Ignoring the polarimetric properties of the scattering problem could lead to inconsistence formulation. Hence, it is important to study the forward scattering behavior of the breast volume numerically. Under this setup with solely breast volumes without any antenna array surrounding it, the scattered field is purely dependent to the breast volume. This allows us to get further understanding about the scattering data in microwave breast imaging problem under different polarization basis at different frequencies. Once we have a good idea about the scattering problem, specific antenna elements and array configurations can be designed. Second, polarization is well treated in the far-field region in frequency domain. Although the corresponding near-field components in the Cartesian coordinates can be computed, the concept of polarization in the near-field region is not well defined. As a result, plane wave incident and scattered far field are considered in this study.

The paper is outlined as follows. An overview of the research problem is given in Sections 2 and 3, followed by some numerical examples in Section 4. Discussions and conclusions will be reached towards the end of the paper.

## 2. Reviews of Microwave Breast Imaging

**Figure 1:**
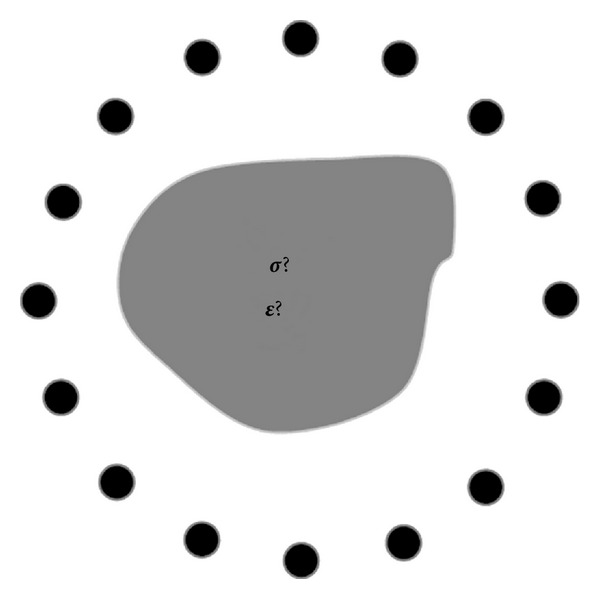
The microwave inverse scattering problem. The object with unknown dielectric properties is surrounded by an array of antenna. The objective is to determine the unknown dielectric properties based on the measured return signals from the antennas [18].

The microwave inverse scattering problem is illustrated in Figure 1. The object with unknown dielectric properties is surrounded by an array of antenna. Similar to X-ray computed tomography (CT), projection of the environment can be obtained by sending electromagnetic waves from one antenna element and receiving the scattered signals from all other antenna elements. By changing the roles of antennas from transmitter to receivers, or rotating the antenna array, multiple views of the unknown object from different angles can be obtained. The objective of microwave inverse scattering problem is to determine for the unknown dielectric profile of the object based on these measured projections.

In this work, we would like to mimic the microwave breast imaging setup as described in [16–18]. The breast volume is placed inside a circular array with 17 monopole antenna elements. The breast volume is illuminated with 1 antenna acting as transmitter and the other antennas acting as receivers such that a projection of the breast volume is obtained. Each antenna element of the array takes turn and the projections of the breast volume from different angles are obtained. In [16–18], the measurement is performed using vector network analyzer (VNA) in frequency domain up to 8 GHz. Depending on the size of the object [16], only a portion of the bandwidth is chosen in frequency domain and the frequency samples are then transformed to the time domain using an inverse Fourier transform followed by a windowing to synthesis a Gaussian amplitude-modulated pulse with sinusoidal carrier. A cost function of the measured electric field *E*_*m*,*n*_^measured^(*t*) and the simulated scattering electric field *E*_*m*,*n*_^simulated^(*t*, *ε*_*r*_(*i*, *j*, *k*), *σ*(*i*, *j*, *k*)) is defined and given by

(1) $$\begin{array}{cc} & F\left(\varepsilon_{r}\left(i,j,k\right),\sigma \left(i,j,k\right)\right)\\ & \;=\overset{T}{\underset{0}{\int }}\overset{M}{\underset{m=1}{\sum }}\overset{N}{\underset{n=1}{\sum }}(|E_{m,n}^{\text{measured}}\left(t\right)\\ & {\;\;\;\;\;\;\;-E_{m,n}^{\text{simulated}}\left(t\cdot \varepsilon_{r}\left(i,j,k\right),\sigma \left(i,j,k\right)\right)|}^{2})dt. \end{array}$$

Here, *ε*_*r*_(*i*, *j*, *k*) are *σ*(*i*, *j*, *k*) the dielectric properties of the assumed breast volume in the simulation environment using FDTD and (*i*, *j*, *k*) is the index of the Yee cell in the FDTD simulation. *M* and *N* are the number of transmitting and receiving antennas, respectively, and the small letters *m* and *n* label the antenna elements. The cost function (1) is minimized using a gradient-based optimization algorithm.

## 3. Forward Scattering of the Breast Volume under Different Polarization States

**Figure 2:**
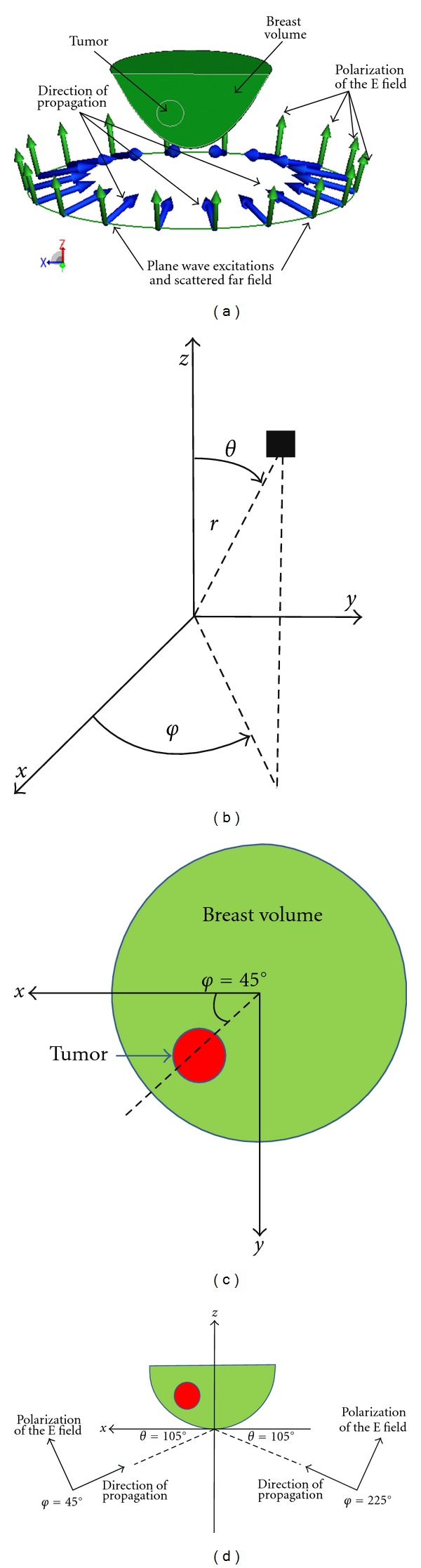
(a)The breast volume under plane wave illumination in FEKO environment. (b) The Spherical coordinate system (*r*, *θ*, *φ*) and the corresponding Cartesian coordinate system (*x*, *y*, *z*) that are used in the FEKO environment. (c) Cross-sectional views (*x*-*y* plane) of the breast volume under plane wave illumination. (d) Cross-sectional view (*φ* = 45°, *θ* = 90°) of the breast volume under plane wave illuminations from *θ*_*t*_ = 105°, *φ*_*t*_ = 45°, and *φ*_*t*_ = 225°, respectively.

**Figure 3:**
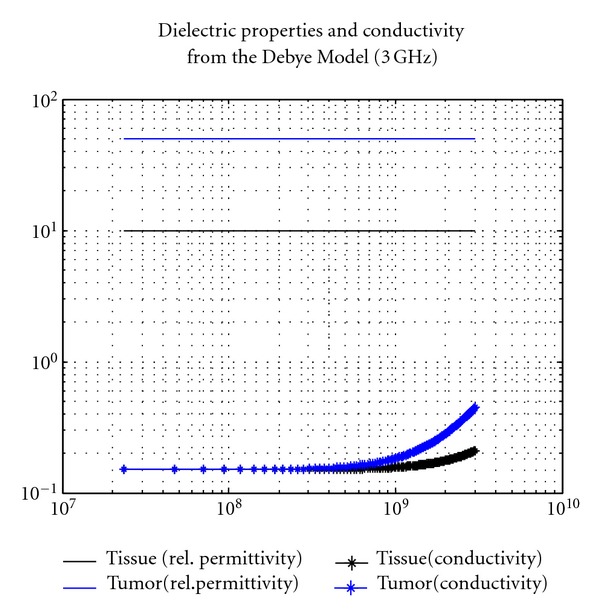
Dielectric Profiles of the breast volume with healthy tissue and tumor using the Debye model. The Debye model is given by *ε*(*f*) = *ε*_*∞*_ − *jσ*/(2*πfε*_0_)+(*ε*_*s*_ − *ε*_*∞*_)/(1 + *jf*/*f*_*p*_), where *ε*_0_ = 8.854 × 10^−12^ F/m, *f*_*p*_ = 25 GHz, and *σ* = 0.15 S/m. For healthy tissue, *ε*_*s*_ = 10 and *ε*_*∞*_ = 7. For the tumor, *ε*_*s*_ = 50 and *ε*_*∞*_ = 35 [3].

In this study, the breast volume shown in Figure 2(a) is considered. It is a hemisphere with radius of 6 cm. The entire breast volume is illuminated from 23 MHz to 3 GHz with 128 samples in frequency domain. The relative permittivity and conductivity of the tissue is given by the widely used Debye model [3, 4] shown in Figure 3. For the case with a tumor inside the breast model, a dielectric sphere centres at the position of (*x* = 15 mm, *y* = 15 mm, *z* = 30 mm, equivalently *r* = 36.7 mm, *θ* = 54.7°, *φ* = 45°) is added. Different sizes of tumor (spheres) with radius of 5 mm, 10 mm, and 15 mm are considered and the dielectric profiles of the tumor can be found in Figure 3. To simulate the breast, imaging scenario without using actual antenna elements, the elevation angle of *θ*_*t*_ = *θ*_*r*_ = 105° is considered (*θ* = 0° corresponds to the positive *z* axis). The subscript *t* and *r* correspond to transmitting and receiving, respectively. The coordinate system and the cross-section view of the breast volume, tumor location, and excitation angle are shown graphically in Figures 2(b) to 2(d). The incident plane wave together with the corresponding scattered far-field at 18 different azimuth directions *φ* are equally spaced within the circle (i.e., Δ*φ* = 20° separation). Both the forward and back scattering directions are considered. For instance, for the plane wave excitation coming from *θ*_*t*_ = 105°, *φ*_*t*_ = 40° (*φ* = 0° corresponds to the positive *x* axis), the scattered far field at all directions (i.e., *θ*_*r*_ = 105°, *φ*_*r*_ = 0°, 20°, 40°, 60°,…, 320°, and 340°) is determined. The computation is done using the commercial hybrid finite element and moment method solver FEKO in the frequency domain [30]. The incident plane wave and scattered far fields under the rectangular and circular polarization basis are considered. For each pair of incidence and scattered directions, the Sinclair polarization matrix of the breast volume is a function of frequency and aspects. This can be given by [31]

(2) $$\begin{array}{cc} \left[S\left(f,\varphi_{t},\varphi_{r}\right)\right] & ={\left[\begin{array}{cc} S_{AA}\left(f,\varphi_{t},\varphi_{r}\right) & S_{AB}\left(f,\varphi_{t},\varphi_{r}\right)\\ S_{BA}\left(f,\varphi_{t},\varphi_{r}\right) & S_{BB}\left(f,\varphi_{t},\varphi_{r}\right) \end{array}\right]}_{\theta_{t}=\theta_{r}=105^{{}^{\circ}}}, \end{array}\;$$

where

(3) $$S_{AB}\left(f,\varphi_{t},\varphi_{r}\right)=\frac{E_{r,B}\left(f,\varphi_{t},\varphi_{r}\right)}{E_{t,A}\left(\text{f},\varphi_{t},\varphi_{r}\right)}.$$

The first subscript (*A* or *B*) in each term in the matrix corresponds to the transmitting polarization state and the second subscript corresponds to the receiving polarization state. Each element *S*_*AB*_(*f*, *φ*_*t*_, *φ*_*r*_) is essentially the ratio of the scattered (received) electric field and the incident (transmitted) electric field. Vertical (V) and horizontal (H) are utilized for linear while left-handed (L) and right-handed (R) are used for circular polarization basis.

## 4. Numerical Results

### 4.1. Monostatic Responses for the Cases with Three Different Tumor Sizes

**Figure 4:**
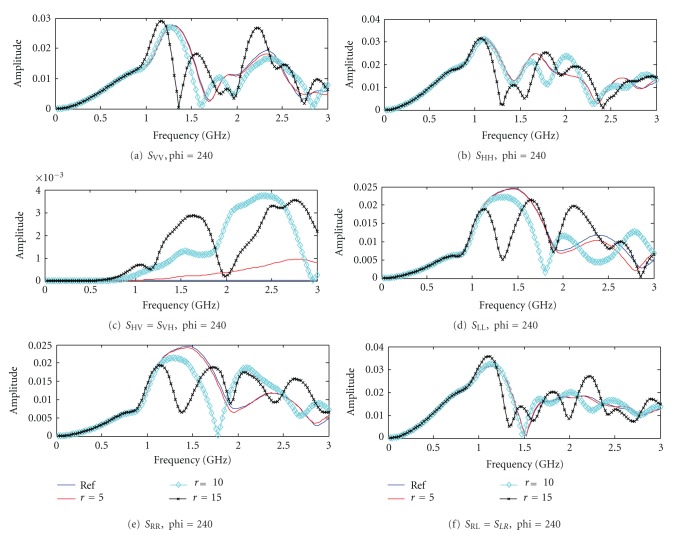
Amplitude response from breast volumes in frequency domain at *θ* = 105°, *φ*_*t*_ = *φ*_*r*_ = 240°. (a) *S*_VV_, (b) *S*_HH_, (c) *S*_HV_ = *S*_VH_, (d) *S*_LL_, (e) *S*_RR_, and (f) *S*_RL_ = *S*_LR_.

As an example, the monostatic amplitude responses of the breast volume at *φ*_*t*_ = *φ*_*r*_ = 240° in frequency domain under different polarization states are shown in Figure 4. Both linear and circular polarization states are considered. Three cases with different radius of the tumors, 5 mm, 10 mm, and 15 mm, are considered and the corresponding amplitude responses are plotted in red, cyan, and black, respectively, in Figure 4. Under monostatic configurations (i.e., *φ*_*t*_ = *φ*_*r*_), *S*_VH_ = *S*_HV_ and *S*_LR_ = *S*_RL_.

**Figure 5:**
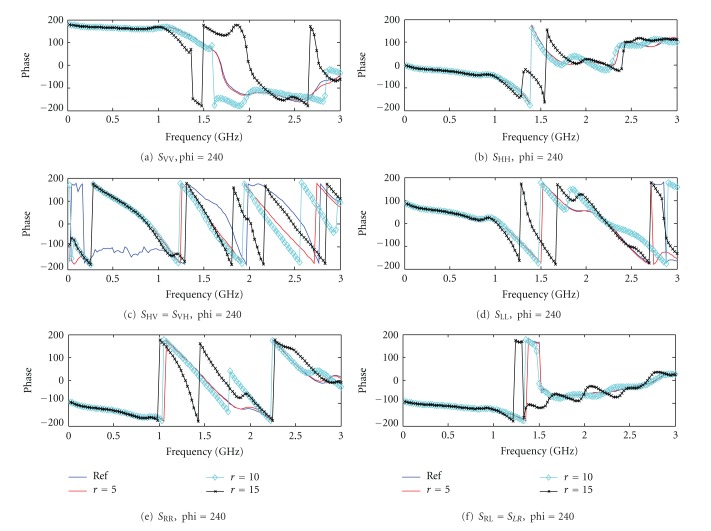
Phase amplitude response from breast volumes in frequency domain at *θ* = 105°, *φ*_*t*_ = *φ*_*r*_ = 240°. (a) *S*_VV_, (b) *S*_HH_, (c) *S*_HV_ = *S*_VH_ (d) *S*_LL_, (e) *S*_RR_, and (f) *S*_RL_ = *S*_LR_.

At frequencies below 1 GHz, it is observed in Figure 4 that the amplitude responses for the four breast volumes are similar for the two linear copolarized and the four circular polarized states. It is apparent that, for the case with 15 mm tumor (black) and 10 mm tumor (cyan), the amplitude response is different to the other two cases when the frequencies are above 1.5 GHz. The results for the case with a 5 mm tumor (red) are almost the same to the reference data (blue) for almost all polarization basis which are difficult to distinguish by visual inspection (except the case VH = HV). It could potentially give a more distinguishable forward dataset for small tumor if VH or HV data is considered. The results indicate that the amplitude responses could be more distinguishable in some polarimetric states than the other, especially in the higher end of the frequency response. Next, the corresponding phase responses as a function of frequency under different polarization states are shown in Figure 5. Similar to the amplitude responses, the phase response for all cases are very similar comparing the reference data (blue) and the 5 mm tumor (red) (except the case VH = HV). The phase responses for the cases with 10 mm (cyan) and 15 mm (black) tumor have more significant differences at above 1 GHz in all cases.

### 4.2. Quantitative Measures as a Function of Transmitting and Receiving Directions

Consider the entire setup that involves a large amount of data (*N*_*tx*_ = *N*_*rx*_ = 18 and 8 polarization states, 18 × 18 × 8 = 2592 sets), visual inspection is not feasible in practice. In view of this, we quantify the differences of the amplitude response between the two cases, that is, breast volume without tumor (reference) and breast volume with a tumor. The relative difference between each frequency sample can be given by

(4) $$\begin{array}{cc} & S_{AB,\text{diff},\text{amplitude}}\left(f,\varphi_{t},\varphi_{r}\right)\\ & \;=\frac{\left|\left|S_{AB,\text{ref}}\left(f,\varphi_{t},\varphi_{r}\right)\right|-\left|S_{AB,\text{tumor}}\left(f,\varphi_{t},\varphi_{r}\right)\right|\right|}{\left|S_{AB,\text{ref}}\left(f,\varphi_{t},\varphi_{r}\right)\right|}\\ & \;\;\times 100\text{\%}. \end{array}$$

A relative measure is chosen here as the scattered electric field varies as the intensity of the incident electric field changes. To quantify the difference of the data at different transmitter/receiver configurations, the “mean differences” of the data at each transmitter and receiver directions, MD_*AB*_, which takes the average from all frequency samples can be given by

(5) $$\begin{array}{cc} & \text{M}{\text{D}}_{AB,\text{amplitude}}\left(\varphi_{t},\varphi_{r}\right)\\ & \;\;=\frac{\left[\sum_{f=\Delta f}^{N\Delta f}S_{AB,\text{diff},\text{amplitude}}\left(f,\varphi_{t},\varphi_{r}\right)\right]}{N}. \end{array}$$

**Figure 6:**
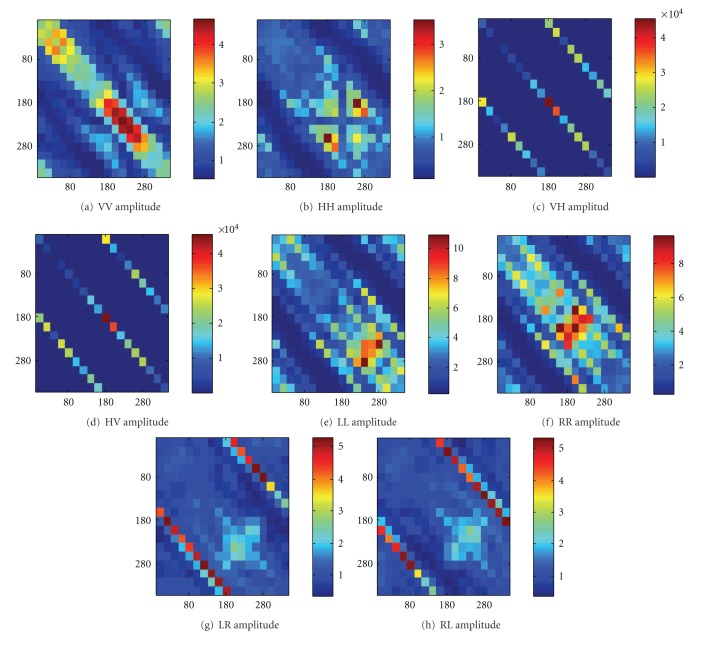
MD_*AB*,amplitude_ for different transmitter/receiver combinations. The comparison is made between the reference data (no tumor) with the case with 5 mm tumor with the dielectric properties given in Figure 3. (a) VV, (b) HH, (c) HV, (d)VH, (e) LL, (f) RR, (g) RL, and (h) LR. The vertical axis corresponds to the aspects of the incidence (*φ*_*t*_ = 0° to 340°) and the horizontal axis corresponds to the aspects of the scattered far field (*φ*_*r*_ = 0° to 340°).

As an example, comparisons between the cases with a 5 mm tumor and the reference data are chosen. This is the case with the smallest differences of the monostatic responses based on the visual inspection when comparing with the reference data. If we could get some insights from this case, it would be more apparent for the other two cases with larger tumors. The results are shown in Figures 6(a) to 6(h). It is observed that MD_*AB*,amplitude_ varies as the transmitter/receiver configurations change. The MD_*AB*,amplitude_s for VV, HH, and the circular polarization basis are less than 4%. More than 1000% of MD_*AB*,amplitude_s are observed for the VH, and HV. Such high values for VH and HV are due to the fact that the cross-polarized component for the case without tumor is almost zero due to the geometrical symmetry. In reality, due to the inhomogeneous nature of human tissue, such high values cannot be achieved. The results under the circular polarization basis are also included. It is shown that the largest MD_*AB*,amplitude_ values for LL and RR (~10%) are higher than those of VV and HH (~5%). According to the heterogeneities shown in Figures 6(e) and 6(f), it seems to show that on average the differences of the forward data under LL and RR polarization are higher than those of the VV and HH. In particular, it is interesting to observe that high values of MD_*AB*,amplitude_ occur at the diagonal axis, which corresponds to the back-scattered direction. For the cross-polarized components (VH, HV, LR, and RL), it is worth noting that the two second diagonals that correspond to the two-dimensional direct path (180° differences in *φ*) also have relative high data contrast. Similarly, comparing Figures 6(g) and 6(h) with Figures 6(a) and 6(b), it seems to show that on average the LR and RL have less differences. To evaluate this properly, another measure will shortly be introduced and we will come back to this later in the next subsection.

Without the loss of generality, similar measures are also made for the phase, given by

(6) $$\begin{array}{cc} & S_{AB,\text{diff},\text{phase}}\left(f,\varphi_{t},\varphi_{r}\right)\\ & \;\;=\left|∠S_{AB,\text{ref}}\left(f,\varphi_{t},\varphi_{r}\right)-∠S_{AB,\text{tumor}}\left(f,\varphi_{t},\varphi_{r}\right)\right|,\\ & \text{M}{\text{D}}_{AB,\text{phase}}\left(\varphi_{t},\varphi_{r}\right)\\ & \;\;=\frac{\left[\sum_{f=\Delta f}^{N\Delta f}S_{AB,\text{diff},\text{phase}}\left(f,\varphi_{t},\varphi_{r}\right)\right]}{N}. \end{array}$$

**Figure 7:**
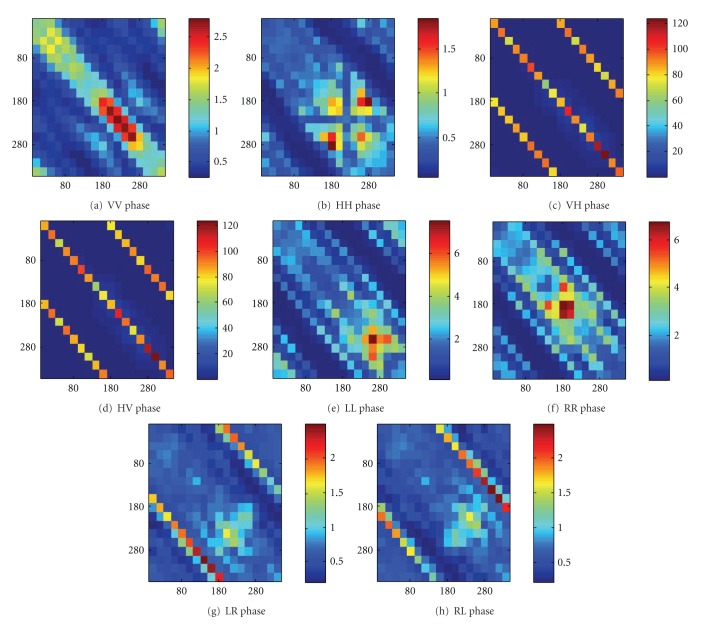
MD_*AB*,phase_ for different transmitter/receiver combinations. The comparison is made between the reference data (no tumor) with the case with 5 mm tumor with the dielectric properties given in Figure 3(b). (a) VV, (b) HH, (c) HV, (d)VH, (e) LL, (f) RR, (g) RL and (h) LR. The vertical axis corresponds to the aspects of the incidence (*φ*_*t*_ = 0° to 340°) and the horizontal axis corresponds to the aspects of the scattered far-field (*φ*_*r*_ = 0° to 340°).

**Figure 8:**
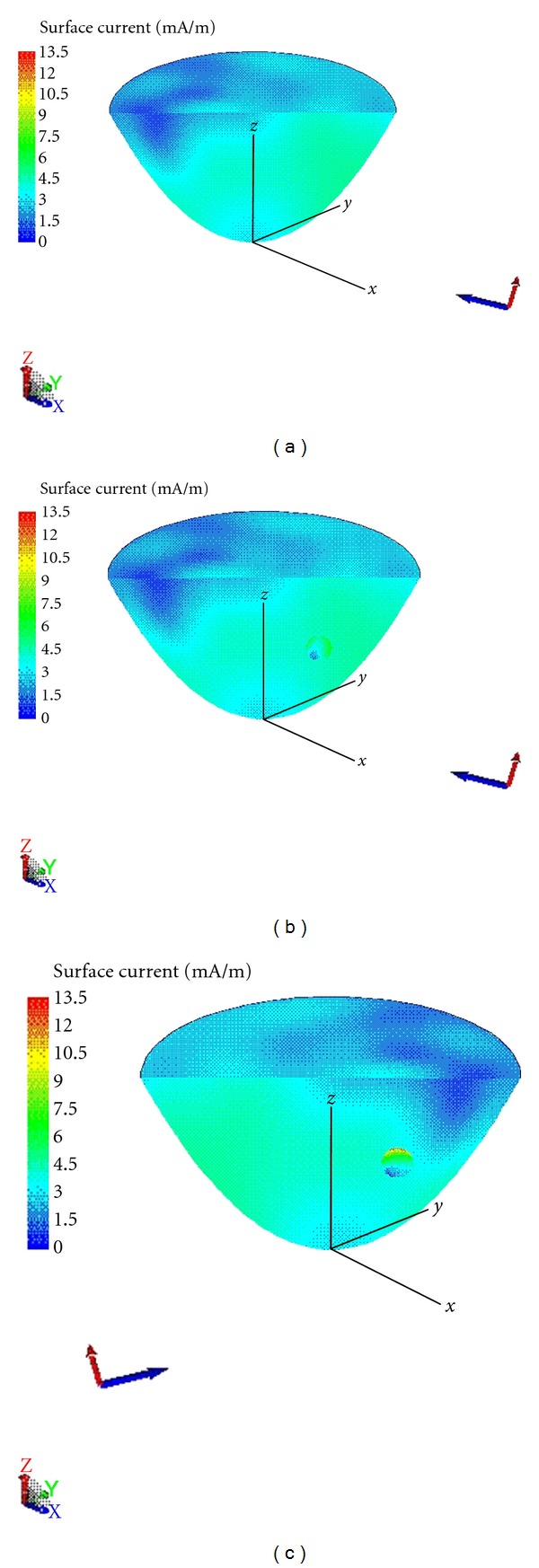
(a) Current distribution of the breast volume under the plane wave excitation with vertical polarized electric field from the aspect of *θ*_*t*_ = 105°, *φ*_*t*_ = 45°. (b) Current distribution of the breast volume with the 5 mm tumor under the plane wave excitation with vertical polarized electric field from the aspect of *θ*_*t*_ = 105°, *φ*_*t*_ = 45°. (c) Current distribution of the breast volume with the 5 mm tumor under the plane wave excitation with vertical polarized electric field from the aspect of *θ*_*t*_ = 105°, *φ*_*t*_ = 225°.

Here, the absolute phase difference is given in terms of degree. For instance, if the two phase angles are 3° and 357°, respectively, *S*_*AB*,diff,phase_(*f*, *φ*_*t*_, *φ*_*r*_) would be equal to 6° instead of 354°. As a result, the maximum and minimum value would be 180° and 0°, respectively. The objective here is to quantify the changes of the phase angle when the tumor is included. The direction of clockwise or anticlockwise is not of interest. The results are shown in Figures 7(a) to 7(h). Similar conclusions can be drawn from the visual inspections of the figures: (i) larger maximum values of MD_*AB*,phase_(*φ*_*i*_, *φ*_*r*_) for LL and RR than VV and HH, (ii) visually the figures look more heterogeneous for LL and RR than VV and HH which probably shows that on average the data contrast of LL and RR is higher (we will come back to it later), (iii) most differences occur along the diagonals which shows that it is important for the back-scattered data, (iv) relatively large contrast also occur at the two second diagonals that correspond to the two-dimensional direct transmission path, (v) significant contrast under VH and HV data. In addition, it is also worth noting that such high values shown in Figures 6 and 7 occur at *φ*_*t*_ = *φ*_*r*_ ≈ 220°–260° but not *φ*_*t*_ = *φ*_*r*_ = 40°–60°, which is where the tumor is located (*φ*_*t*_ = *φ*_*r*_ = 45°). To understand this, the breast volume under the two excitations with vertically polarized electric field and the corresponding current distributions at 2 GHz are shown in Figures 8(a) to 8(c). The problem is first shown in Figure 2(d). For the case without a tumor, the breast volume is symmetric and thus the current distribution under the excitation of *φ* = 45° is a mirror image of that under the excitation of *φ* = 225°. The current distributions for the cases of (i) without the tumor under the excitation of *θ* = 105°, *φ* = 45°, with the 5 mm tumor under the excitation of (ii) *θ* = 105°, *φ* = 45°, and of (iii) *θ* = 105°, *φ* = 225° are shown in Figures 8(a) to 8(c), respectively. For a fair visual comparison, the figures are set to the same intensity scale from 0 mA/m to 13.50 mA/m. When the breast volume is illuminated from *θ* = 105°, *φ* = 45°, it is observed that the maximum amplitude of the induced current is increased when the tumor is introduced. In particular, the maximum current amplitude is located at the tumor with ~7.5 mA/m. When the excitation changes to *θ* = 105°, *φ* = 225°, the maximum current is again located at the tumor and is now increased to ~13.50 mA/m. Comparing the current distribution of the tumor under the two different excitation, on average the current distribution is higher in the latter case which shows that the tumor is better illuminated. The above observation can be explained by the fact that the complexity of the scattering phenomena inside the breast volume. First, the breast surface is curative and the reflection coefficient (air-breast volume) varies as a function of incident angles, frequency and polarization. Due to the curative nature of the breast surface, the breast volume is not evenly illuminated even under plane wave illumination. Second, the higher-order electromagnetic interactions between the tumor and the breast volume (tumor-breast interface) and the air-breast interface could help focusing the energy toward the tumor. In general, such higher-order interactions are complicated and cannot be easily analyzed. To sum up, using full-wave electromagnetic simulation, it is found that the larger amplitude of the induced current on the tumor and the better data contrast of the copolarized response are resulted under the illumination of *θ* = 105°, *φ* = 225°.

### 4.3. Cases with Tumors with Lower Contrast

A large-scale study about experimental measurements of dielectric properties of tumors has been conducted and reported in [26, 27]. The results have shown that the dielectric properties are far much lower than the earlier findings with 300% or even 500% of contrast. With this in mind, we would like to investigate how the contrast could affect the forward data. Here, tumors with relative permittivity of *ε*_*r*_ = 40 down to *ε*_*r*_ = 15, with the conductivity of *σ* = 0.1 S/m, together with the previous cases, will be considered.

In order to get an overall picture about the differences of the amplitude and phase response for each polarization states, the mean values of MD_*xy*_, denoted as MMD_*xy*_, can be given by

(7) $$\begin{array}{cc} \text{MM}{\text{D}}_{AB,\text{amplitude}} & =\frac{\left[\sum_{\phi_{r}=0^{{}^{\circ}}}^{340^{{}^{\circ}}}\sum_{\phi_{i}=0^{{}^{\circ}}}^{340^{{}^{\circ}}}\text{M}{\text{D}}_{AB,\text{amplitude}}\left(\varphi_{t},\varphi_{r}\right)\right]}{\left[N_{tx}\times N_{rx}\right]},\\ \text{MM}{\text{D}}_{AB,\text{phase}} & =\frac{\left[\sum_{\phi_{r}=0^{{}^{\circ}}}^{340^{{}^{\circ}}}\sum_{\phi_{i}=0^{{}^{\circ}}}^{340^{{}^{\circ}}}\text{M}{\text{D}}_{AB,\text{phase}}\left(\varphi_{t},\varphi_{r}\right)\right]}{\left[N_{tx}\times N_{rx}\right]}. \end{array}$$

**Figure 9:**
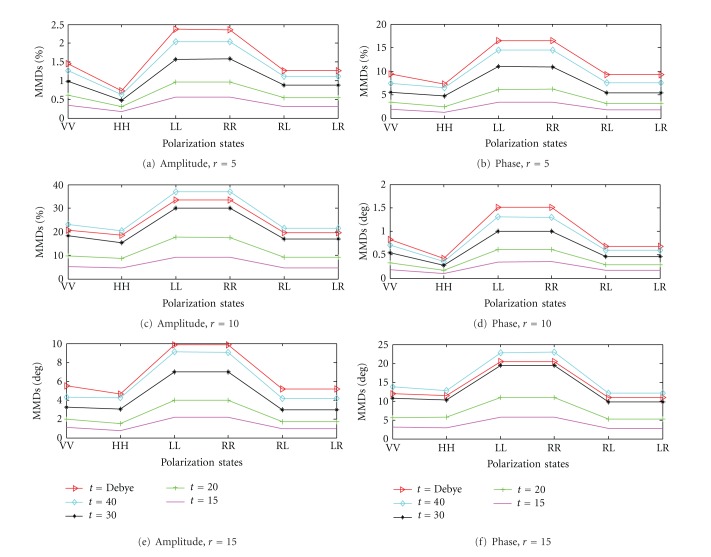
MMD_*AB*_ for different polarization states with tumor of different sizes. In each figure, three different dielectric properties of the tumor are considered (i) Debye (Figure 3), (ii) *ε*_*r*_ = 40, *σ* = 0.1 S/m, (iii) *ε*_*r*_ = 30, *σ* = 0.1 S/m, (iv) *ε*_*r*_ = 20, *σ* = 0.1 S/m, and (v) *ε*_*r*_ = 15, *σ* = 0.1 S/m. MMD_*AB*,amplitude_ for tumor with radius of (a) 5 mm, (b) 10 mm, and (c) 15 mm. MMD_*AB*,phase_ for tumor with radius of (d) 5 mm, (e) 10 mm, and (f) 15 mm.

**Figure 10:**
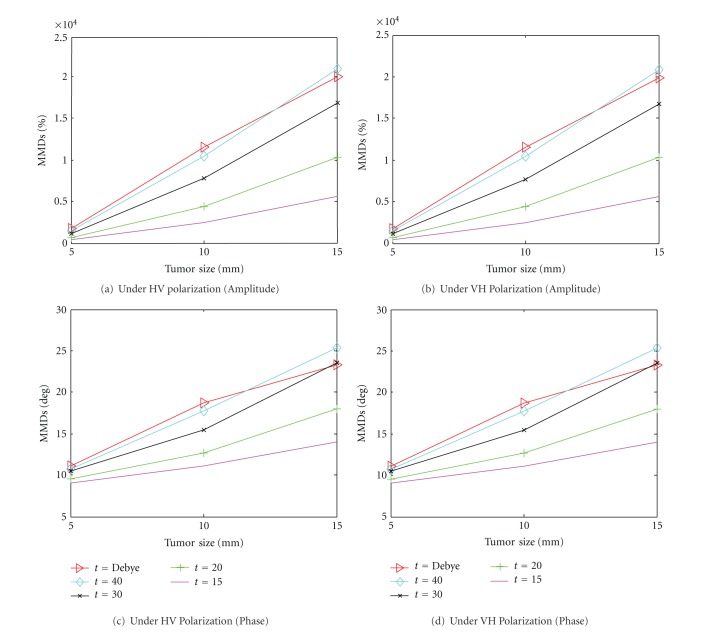
MMD_*AB*,amplitude_ and MM*i*_*AB*,phase_ for the scattering problems with tumor of different sizes. In each figure, three different dielectric properties of the tumor are considered (i) Debye (Figure 3), (ii) *ε*_*r*_ = 40, *σ* = 0.1 S/m, (iii) *ε*_*r*_ = 30, *σ* = 0.1 S/m, (iv) *ε*_*r*_ = 20, *σ* = 0.1 S/m, and (v) *ε*_*r*_ = 15, *σ* = 0.1 S/m. (a) MMD_*AB*,amplitude_ for HV polarization state, (b) MMD_*AB*,amplitude_ for VH polarization state, (c) MMD_*AB*,phase_ for HV polarization state, (d) MMD_*AB*,phase_ for VH polarization state.

The corresponding results are shown in Figures 9(a) and 9(f).  Figure 9(a) shows the MMD_*AB*,amplitude_ for the cases with 5 mm tumor (*r* = 5) but with different dielectric properties of (i) Debye, (ii) *ε*_*r*_ = 40, *σ* = 0.1 S/m, (iii) *ε*_*r*_ = 30, *σ* = 0.1 S/m, (iv) *ε*_*r*_ = 20, *σ* = 0.1 S/m, and (v) *ε*_*r*_ = 15, *σ* = 0.1 S/m. As the contrast increases, higher values of MMD_*AB*,amplitude_s result which indicates that contrast does play a role here. It is interesting to see that the increase of the dielectric contrast gives a proportional change of the data contrast for the same tumor size. For instance, as the relative permittivity changes from 30 to 40, the MMD_*xy*,amplitude_ values for the 5 mm tumor (Figure 9(a)) and 10 mm tumor (Figure 9(b)) increase from 1.5% to 2% and 10% to 15% (about one third) for LL and RR polarization states. However, the maximum data contrast that can be obtained for the 5 mm tumor is less than 2.5% with the maximum dielectric contrast of approximately 5. Next, we consider the results for the 10 mm tumor (*r* = 10) and the 15 mm tumor (*r* = 15) shown in Figures 9(b) and 9(c), respectively. As the tumor size increases, MMD_*xy*,amplitude_ values increase from <2.5% to ~15% and ~30%, respectively. This again shows that the contrast is more significant with larger tumor. The results here indicate that the tumor size plays a relatively more important role in terms of the “contrast of the forward data” than the dielectric contrast of the tumor with the background. If we consider the case of a 5 mm tumor (Figure 9(a)), increasing the contrast from 1.5 times (*ε*_*r*,tumor_/*ε*_*r*,tissue_ ≈ 15/10 = 1.5) to 5 (*ε*_*r*,tumor_/*ε*_*r*,tissue_ ≈ 50/10 = 5) times could perhaps raise increasing the data contrast from 0.5% to 2%, but increasing the tumor size (from 5 mm to 15 mm radius) can significantly increase the contrast to up to 10%. Similar conclusions can also be drawn for the phase responses shown in Figures 9(d) to 9(f).

Compare to our previous visual observations, for the same tumor size, the results from the LL and RR polarization states have larger MMD_*AB*,amplitude_ and MMD_*AB*,phase_ values than the other polarization states which indicates higher level of contrast in the forward data. For the 5 mm tumor, VV has got a larger MMD_*AB*,amplitude_ and MMD_*AB*,phase_ than those of HH, VH, HV, RL, and LR. Such findings further confirm the observations we had made earlier.

The results for VH and HV are plotted separately in Figure 10 as the values of MMD_*AB*,amplitude_ are much higher than the other polarization states, ranging from 100% up to 2000%. Such high values of MMD_*AB*,amplitude_ are due to the fact that VH and HV responses are theoretically zero due to the geometrical symmetric feature of the breast volume when there is no tumor.  Figure 10(a) shows the MMD_*AB*,amplitude_ values under HV polarization state. The horizontal axis corresponds to the tumor size and the three lines correspond to tumor with different dielectric properties. Similar to the previous findings, the MMD_*AB*,amplitude_ values increase with the tumor size. Increasing the dielectric contrast could increase the contrast of the forward data, but for small tumor (5 mm), increasing the dielectric contrast does not give much changes to the dataset with relatively small variations of MMD_*AB*,amplitude_ and MMD_*AB*,phase_ shown in Figures 10(a) and 10(b). The same conclusions are drawn for HV polarization states, as shown in Figures 10(c) and 10(d).

### 4.4. Feasibility Measures in Terms of Signal Level

The above findings show that the higher contrast of the forward data can be obtained under the copolarized case for circular polarization basis and cross-polarized cases for linear polarization basis. The next question we have to answer is if it is possible to measure the signal, especially for the VH and HV cases as the signal level could be very low (the high MMD_*AB*,amplitude_ values are due to the null response when there is no tumor). In view of this, the mean and minimum values of the scattering parameters as a function of frequency are introduced and given by

(8) $$\begin{array}{cc} S_{AB,\text{mean}}\left(f\right) & =\frac{\left[\sum_{\phi_{r}=0^{{}^{\circ}}}^{340^{{}^{\circ}}}\sum_{\phi_{i}=0^{{}^{\circ}}}^{340^{{}^{\circ}}}20\text{lo}{\text{g}}_{10}\left|S_{AB}\left(f,\phi_{t},\phi_{r}\right)\right|\right]}{\left[N_{tx}\times N_{rx}\right]}\\ & =\frac{\left[\sum_{\phi_{r}=0^{{}^{\circ}}}^{340^{{}^{\circ}}}\sum_{\phi_{i}=0^{{}^{\circ}}}^{340^{{}^{\circ}}}20\text{lo}{\text{g}}_{10}\left|\frac{E_{r,B}\left(f,\phi_{t},\phi_{r}\right)}{E_{t,A}\left(f,\phi_{t},\phi_{r}\right)}\right|\right]}{\left[N_{tx}\times N_{rx}\right]},\\ S_{AB,\min}\left(f\right) & =\min\left\{20\text{lo}{\text{g}}_{10}\left|S_{AB}\left(f,\phi_{i},\phi_{r}\right)\right|\right\}\\ & =\min\left\{20\text{lo}{\text{g}}_{10}\left|\frac{E_{r,B}\left(f,\phi_{i},\phi_{r}\right)}{E_{t,A}\left(f,\phi_{i},\phi_{r}\right)}\right|\right\}. \end{array}$$

**Figure 11:**
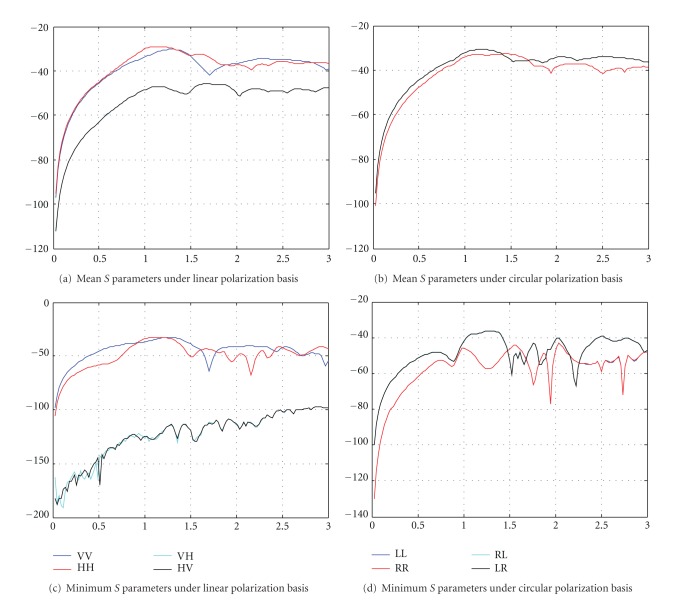
*S*_*AB*,mean_(*f*) and *S*_*AB*,min⁡_(*f*) for the scattering problem of the breast volume with a 5 mm tumor with the dielectric properties of *ε*_*r*_ = 15, *σ* = 0.1 S/m. (a) *S*_*AB*,mean_(*f*) for linear polarization states, (b) *S*_*AB*,mean_(*f*) for circular polarization states, (c) *S*_*AB*,min⁡_(*f*) for linear polarization states and (d) *S*_*AB*,min⁡_(*f*) for circular polarization states.

The results shown in Figures 11(a) to 11(d) are the case of the breast volume with the 5 mm tumor with the properties of *ε*_*r*_ = 15, *σ* = 0.1 S/m, that is, the case with the smallest tumor and lowest dielectric contrast. For both linear and circular polarization states, the mean values of the scattering parameters are within 60 dB dynamic range when the frequency is above 500 MHz. The minimum values of the scattering parameters are shown in Figures 11(c) and 11(d). The results show that the minimum values of the scattering parameters are within −80 dB below the transmitting signal for circular polarization states, as well as VV and HH (above 500 MHz). For VH and HV, however, it goes far below −100 dB which could be difficult to measure. With the current state-of-the-art of VNA, we are able to measure signals down to −130 dB accurately. In practice, however, together with practical considerations such as antenna mismatch and cable loss, the signals level would be at least another 10 dB to 15 dB lower. As a result, accurate measurement of VH and HV signals for breast volumes with small tumors is not easy to achieve using VNA. Proper design of the receiving modules with matching antenna and front-end electronics becomes significantly crucial. On the other hand, it would be feasible to measure the circular polarized signals using VNA (>500 MHz, minimum values between −60 dB to −80 dB + another 20 dB losses for mismatches).

## 5. Discussions and Conclusions

UWB forward scattering data from breast volumes with different tumor sizes and different dielectric properties are studied. Based on the configurations of the forward scattering study in this paper, several points can be summarized. To achieve good contrast of the amplitude and phase responses of the forward data between the cases with and without tumor, the excitation frequency should be at least 1 GHz as the lower-frequency components correspond mainly to the scattering from the entire breast volume. At the same time, the return signal level is relative low when the frequency is below 500 MHz. This shows that the breast volume is not well excited. Secondly, it is also found that there are higher contrast of the scattering data in the back-scattered direction for all cases and the direct path for cross polarized cases. This implies the importance of having the back scattered field and the direct path response in the microwave imaging setup. Thirdly, comparing the forward scattering data under different polarization states and basis, VH and HV components have the highest contrast due to the geometrical symmetry when there is no tumor inside the breast volume. Regarding other polarization states, the LL and RR polarization states give better data contrast than the others. Potentially, LL and RR can be used for microwave imaging using existing VNA that can give reasonable accuracies with more than 100 dB of dynamic range. If one would like to use VH and HV, proper design of front-end microwave circuits and antenna are needed such that the mismatch can be minimized.

This work opens the door for further investigations of better datasets for the microwave breast imaging by considering different polarization states and basis. Although the setup is relatively simple with homogenous breast volume, surprisingly it is found that the existence of small tumor (*r* = 5 mm) is not highly revealed in the forward data even if the dielectric contrast is about 5 times. Potentially, better data contrast could be obtained for small tumor with higher excitation frequency, but at the same time attenuation increases. Our previous study on a similar problem has found that the conductivity of human tissue can significantly attenuate the higher-order interactions of metallic objects inside human tissue when the excitation frequency goes beyond 4 GHz [32–35]. As a result, higher excitation frequencies were not considered in this work. Future work needs to focus on practical issues such as the choice of polarization states, antenna elements, array configurations, matching liquid, and more realistic tissue model in the simulation [26, 27]. At the same time, the results here for VH and HV only apply for homogenous breast models. To investigate the feasibilities of using linear cross-polarized signals, we also need to use anatomically and electromagnetically realistic breast model in future simulations.

## References

[1] Fear EC, Stuchly MA (2000). Microwave detection of breast cancer. *IEEE Transactions on Microwave Theory and Techniques*.

[2] Fear EC, Hagness SC, Meaney PM, Okoniewski M, Stuchly MA (2002). Enhancing breast tumor detection with near-field imaging. *IEEE Microwave Magazine*.

[3] Hagness SC, Taflove A, Bridges JE (1998). Two-dimensional FDTD analysis of a pulsed microwave confocal system for breast cancer detection: fixed-focus and antenna-array sensors. *IEEE Transactions on Biomedical Engineering*.

[4] Li X, Hagness SC (2001). A confocal microwave imaging algorithm for breast cancer detection. *IEEE Microwave and Wireless Components Letters*.

[5] Fear EC, Li X, Hagness SC, Stuchly MA (2002). Confocal microwave imaging for breast cancer detection: localization of tumors in three dimensions. *IEEE Transactions on Biomedical Engineering*.

[6] Li X, Davis SK, Hagness SC, Van Der Weide DW, Van Veen BD (2004). Microwave imaging via space-time beamforming: experimental investigation of tumor detection in multilayer breast phantoms. *IEEE Transactions on Microwave Theory and Techniques*.

[7] Li X, Bond EJ, Van Veen BD, Hagness SC (2005). An overview of ultra-wideband microwave imaging via space-time beamforming for early-stage breast-cancer detection. *IEEE Antennas and Propagation Magazine*.

[8] Sill JM, Fear EC (2005). Tissue sensing adaptive radar for breast cancer detection: study of immersion liquids. *Electronics Letters*.

[9] Sill JM, Fear EC (2005). Tissue sensing adaptive radar for breast cancer detection-experimental investigation of simple tumor models. *IEEE Transactions on Microwave Theory and Techniques*.

[10] Khor WC, Bialkowski ME, Abbosh A, Seman N, Crozier S (2007). An ultra wideband microwave imaging system for breast cancer detection. *IEICE Transactions on Communications*.

[11] Nilavalan R, Gbedemah A, Craddock IJ, Li X, Hagness SC (2003). Numerical investigation of breast tumour detection using multi-static radar. *Electronics Letters*.

[12] Klemm M, Craddock IJ, Leendertz JA, Preece A, Benjamin R (2009). Radar-based breast cancer detection using a hemispherical antenna array—experimental results. *IEEE Transactions on Antennas and Propagation*.

[13] Fang Q, Meaney PM, Geimer SD, Streltsov AV, Paulsen KD (2004). Microwave image reconstruction from 3-D fields coupled to 2-D parameter estimation. *IEEE Transactions on Medical Imaging*.

[14] Rubæk T, Meaney PM, Meincke P, Paulsen KD (2007). Nonlinear microwave imaging for breast-cancer screening using Gauss-Newton’s method and the CGLS inversion algorithm. *IEEE Transactions on Antennas and Propagation*.

[15] Rubæk T, Kim OS, Meincke P (2009). Computational validation of a 3-D microwave imaging system for breast-cancer screening. *IEEE Transactions on Antennas and Propagation*.

[16] Fhager A, Hashemzadeh P, Persson M (2006). Reconstruction quality and spectral content of an electromagnetic time-domain inversion algorithm. *IEEE Transactions on Biomedical Engineering*.

[17] Fhager A, Persson M (2007). Using a priori data to improve the reconstruction of small objects in microwave tomography. *IEEE Transactions on Microwave Theory and Techniques*.

[18] Fhager A (2006). *Microwave tomography*.

[19] D. Shea J, Kosmas P, C. Hagness S, D. Van Veen B (2010). Contrast-enhanced microwave imaging of breast tumors: a computational study using 3D realistic numerical phantoms. *Inverse Problems*.

[20] Shea JD, Kosmas P, Hagness SC, Van Veen BD (2010). Three-dimensional microwave imaging of realistic numerical breast phantoms via a multiple-frequency inverse scattering technique. *Medical Physics*.

[21] Zhang J, Fear EC Preliminary investigation of breast tumor detection using cross-Vivaldi antenna.

[22] Noghanian S, Craddock I Microwave imaging’s practical issues.

[23] Zeng X, Fhager A, Persson M, Linner P, Zirath H (2011). Accuracy evaluation of ultra-wideband time domain measurement systems for microwave imaging. *IEEE Transactions on Antennas and Propagation*.

[24] Zeng X, Fhager A, Linner P, Persson M, Zirath H (2011). Experimental investigation of the accuracy of an ultra-wideband time domain microwave tomography system. *IEEE Transactions on Instrumentation and Measurement*.

[25] Zhang ZQ, Liu QH, Xiao C, Ward E, Ybarra G, Joines WT (2003). Microwave breast imaging: 3-D forward scattering simulation. *IEEE Transactions on Biomedical Engineering*.

[26] Lazebnik M, McCartney L, Popovic D (2007). A large-scale study of the ultrawideband microwave dielectric properties of normal breast tissue obtained from reduction surgeries. *Physics in Medicine and Biology*.

[27] Lazebnik M, Popovic D, McCartney L (2007). A large-scale study of the ultrawideband microwave dielectric properties of normal, benign and malignant breast tissues obtained from cancer surgeries. *Physics in Medicine and Biology*.

[28] Pastorino M (2010). *Microwave Imaging*.

[29] Boerner WM, El-Arini MB, Chan CY, Mastoris PM (1981). Polarization dependence in electromagnetic inverse problems. *IEEE Transactions on Antennas and Propagation*.

[30] FEKO EM Software & Systems S.A.

[31] Mott H (2007). *Remote Sensing with Polarmetric Radar*.

[32] Lui HS, Shuley NVZ (2007). Detection of depth changes of a metallic target buried in a frequency-dependent lossy halfspace using the E-pulse technique. *IEEE Transactions on Electromagnetic Compatibility*.

[33] Lui HS, Shuley NVZ, Rakic AD (2010). A novel, fast, approximate target detection technique for metallic target below a frequency dependant lossy halfspace. *IEEE Transactions on Antennas and Propagation*.

[34] Lui HS, Shuley N, Persson M Joint time-frequency analysis of transient electromagnetic scattering from a subsurface target.

[35] Lui HS, Aldhubaib F, Shuley NVZ, Hui HT (2009). Subsurface target recognition based on transient electromagnetic scattering. *IEEE Antennas and Propagation Magazine*.

